# Motion behaviour recognition dataset collected from human perception of collective motion behaviour

**DOI:** 10.1016/j.dib.2023.108976

**Published:** 2023-02-15

**Authors:** Shadi Abpeikar, Kathryn Kasmarik

**Affiliations:** School of Engineering and IT, UNSW Canberra, Northcott Drive, Canberra, ACT, 2612, Australia

**Keywords:** Swarming, Online survey, Binary supervised dataset, Supervised machine learning, Boids

## Abstract

Collective motion behaviour such as the movement of swarming bees, flocking birds or schooling fish has inspired computer-based swarming systems. They are widely used in agent formation control, including aerial and ground vehicles, teams of rescue robots, and exploration of dangerous environments with groups of robots. Collective motion behaviour is easy to describe, but highly subjective to detect. Humans can easily recognise these behaviours; however, it is hard for a computer system to recognise them. Since humans can easily recognise these behaviours, ground truth data from human perception is one way to enable machine learning methods to mimic this human perception. Hence ground truth data has been collected from human perception of collective motion behaviour recognition by running an online survey. In this survey, participants provide their opinion about the behaviour of ‘boid’ point masses. Each question of the survey contains a short video (around 10 seconds), captured from simulated boid movements. Participants were asked to drag a slider to label each video as either ‘flocking’ or ‘not flocking’; ‘aligned’ or ‘not aligned’ or ‘grouped’ or ‘not grouped’. By averaging these responses, three binary labels were created for each video. This data has been analysed to confirm that it is possible for a machine to learn binary classification labels from the human perception of collective behaviour dataset with high accuracy.

**Specifications Table**

**Table:** 

Subject	Artificial Intelligence
Specific subject area	The database is useful for automatic collective behaviour recognition using supervised machine learning methods in the field of swarm intelligence.
Type of data	Table of data records comprising numerical features.
How the data were acquired	The database has been collected from an online survey. The online survey shows 16 short video clips to participants as 16 ‘questions’. Each video is shows point mass ‘boids’ moving around in a simulated environment. The participants must vote for these videos with three sliders: “flocking-not flocking”, “aligned-not aligned “, and “group-not grouped”. The participants put the slider point closer to the term which is most similar to their opinion of the presented video. We then extracted features from each video and processed participant responses to generate labels for these features. The screenshots from the online survey are available in the Supplementary Materials.
Data format	Raw
Description of data collection	90 participants answered the online survey of human perceptions of collective behaviour. They were from all over the world, each over 18 years old, with diverse levels of knowledge of collective behaviour. The only requirement was that they could access the link to the survey, agree to the participant information statement and submit the webform, from anywhere with no need to be in a specific lab. The class label of each record is the average over all participant responses.
Data source location	Raw data stored at: • Institution: University of New South Wales, Canberra • City/Town/Region: Canberra, ACT • Country: Australia
Data accessibility	Repository name: Harvard Dataverse Data identification number: https://doi.org/10.7910/DVN/S1YJOX Direct URL to data: Replication Data for: Automatic Collective Behaviour Recognition - Harvard Dataverse
Related research article	Kasmarik, K., Abpeikar, S., Khan, M. M., Khattab, N., Barlow, M., & Garratt, M. (2020, November). Autonomous recognition of collective behaviour in robot swarms. In Australasian Joint Conference on Artificial Intelligence (pp. 281-293). Springer.

## Value of the Data

• **Why is this data useful?** Humans can recognize collective motion behaviours very easily, while it is a difficult task for machines. In fact, no set of objective measures exist by which a machine can identify all kinds of collective motion. Collecting a dataset that captures human perception of collective motion will help build or train machines that can automatically recognise these behaviours, by mimic human perception of collective motion.
• **Who can benefit?** This data will help industries that want to use swarming robots to recognize and control swarming behaviours automatically. It can also benefit machine learning researchers or researchers in swarm intelligence who would like a ground truth labelling of collective motion behaviours.
• **How can the data be used?** This data can be used to train supervised learning algorithms to mimic human perception of swarming motion. Trained learners can then be embedded in secondary learning systems to tune or evolve swarming behaviour.
• **Future uses:** In future we envisage that it may be possible to apply transfer learning from this dataset to control swarming robots with a variety of behavioural dynamics.

## 1 Objective

Collective motion behaviour refers to the way groups of birds, animals or insects move in nature. Although simulating collective motion behaviour to solve abstract problems is important, recognising this behaviour is vital too. Recognition of collective behaviour has potential application in areas such as civil aviation [1], defence [2], [3], [4], swarm robotics [5], [6], [7] and human-swarm interaction missions where humans control groups of unmanned vehicles [8]. It is relatively easy to simulate behaviour that humans will recognize as collective behaviour. However, it remains difficult to build computer systems that can recognize the emergence of these diverse collective behaviours in artificial agents. One immediate solution is to take advantage of human ability by collecting data that captures the human perception of collective behaviour recognition. The aim of this data collection is to provide training data from which a machine can learn to imitate human perception and recognition of diverse, artificial, collective motions. This dataset will allow researchers to build systems to recognise different aspects of collective motion. This will extend existing work that has focused on recognition of flocking and not-flocking motion [6].

## 2 Data Description

**Fig. 1:**
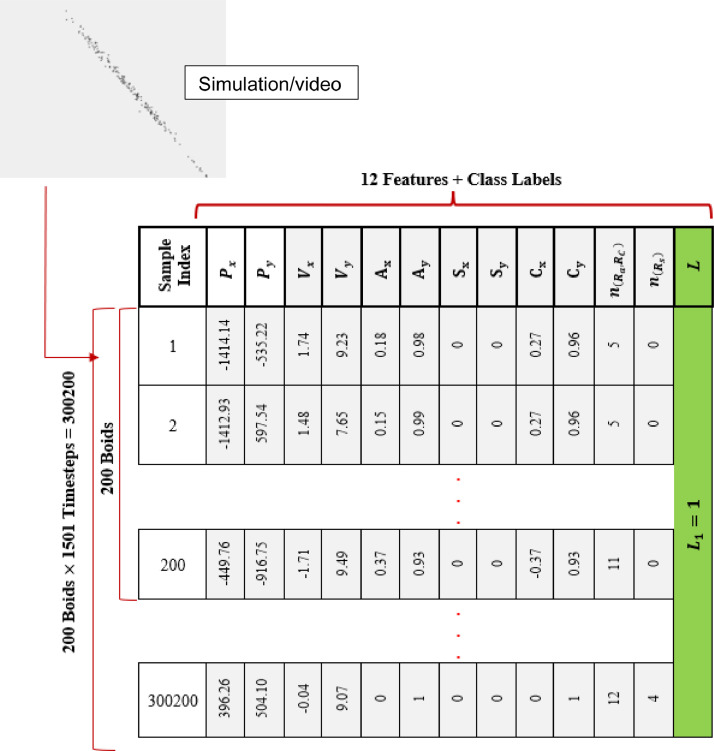
Structure of the flocking dataset for one of the 16 behaviours (class $L_{1}$).

The structure of this dataset is illustrated in Fig. 1. This dataset is an adaptation of the data provided in [9] that reorganises and filters the data in an appropriate way for use in machine learning. The dataset was created in three steps: (1) simulating point mass boids engaged in collective motion; (2) an online survey to gather human labels for video clips from these simulations; and (3) extraction of features from each video and assignment of labels. Steps (1) and (3) are described in this section and Step (2) in the next section.

### 2.1 Computer Simulation of Collective Motion

The database includes 16 behaviours, and the features of the records in this dataset were generated using a boid model [10] augmented with a number of situational awareness (SA) parameters in a point mass simulator [11]. The three fundamental rules of Reynolds’ boid model are Cohesion (a boid should move towards the average position of its neighbouring boids); Alignment (a boid should steer to align itself with the average heading of its neighbouring boids); and Separation (a boid should move to avoid collision with neighbouring boids). These rules are implemented as forces that act on point mass boids. Suppose we have a group of N boids $B^{1},\;B^{2},\;B^{3}\ldots .,\;B^{N}$. At time t each boid $B^{i}$ has a position point $P_{t}^{i}$, and a velocity vector$\;v_{t}^{i}$. At each timestep t, the velocity of each boid is updated as follows:

(1) $$\;v_{t+1}^{i}=\;v_{t}^{i}+W_{c}\;c_{t}^{i}+W_{a}\;a_{t}^{i}+\;W_{s}s_{t}^{i}$$

$c_{t}^{i}$ is a vector in the direction of the average position of boids within a radius $R_{c}$ of $B^{i}$ (called the neighbours of $B^{i}$); $a_{t}^{i}$ is a vector in the average direction of boids within a radius $R_{a}$ of $B^{i}$; and $s_{t}^{i}$ is a vector in the direction away from the average position of boids within a radius $R_{s}$ of $B^{i}$. To attain $c_{t}^{i}$, $a_{t}^{i}$, and $s_{t}^{i}$, we need to define a subset $N^{i}$ of agents within a certain range of $R\in \{R_{c}$, $R_{a}$, $R_{s}\}$ of $A^{i}$ as follows, where the Euclidean distance between two agents $A^{k}$, and $A^{i}$ is computed by $\text{dist}(A^{k},A^{i})$:

(2) $$N^{i}=\left\{A^{k}|A^{k}\neq A^{i}\wedge \text{dist}\left(A^{k},A^{i}\right)<R\right\}$$

Therefore, the average ${\vec{c}}_{t}^{i}$ of agents within range $R_{c}\;$of $A^{i}$ can be calculated by Eq. (3), where $x_{t}^{k}$ is the current position of boids.

(3) $${\vec{c}}_{t}^{i}=\frac{\sum_{k}x_{t}^{k}}{\left|{\left(N_{c}\right)}_{t}^{i}\right|}\;$$

By using Eq. (3), the vector in the direction of ${\vec{c}}_{t}^{i}$ can be calculated as follows:

(4) $$c_{t}^{i}={\vec{c}}_{t}^{i}-x_{t}^{i}\;$$

Similarly, if we consider $N_{s}$ as the set of agents in the range $R_{s}$ of $A^{i}$, the average ${\vec{s}}_{t}^{i}$ of agents within this range can be calculated as Eq. (5).

(5) $${\vec{s}}_{t}^{i}=\frac{\sum_{k}x_{t}^{k}}{\left|{\left(N_{s}\right)}_{t}^{i}\right|}\;$$

Therefore, $s_{t}^{i}$ as the vector away from ${\vec{s}}_{t}^{i}$ is calculated as follows:

(6) $$s_{t}^{i}=x_{t}^{i}-{\vec{s}}_{t}^{i}\;$$

Then, the average direction of agents within the range $R_{a}\;$of $A^{i}$, the vector $a_{t}^{i}$ is calculated as follows:

(7) $$a_{t}^{i}=\frac{\sum_{k}v_{t}^{k}}{\left|{\left(N_{a}\right)}_{t}^{i}\right|}\;$$

Weights $W_{c},W_{a}$ and $W_{s}$ strengthen or weaken the corresponding force in Eq. (1). The newly calculated velocity is further normalized and scaled by a speed value chosen between a maximum $V_{\text{max}}$ and minimum $V_{\text{min}}$. Once a new velocity has been computed, the position of each boid is updated by Eq. (8).

(8) $$P_{t+1}^{i}=P_{t}^{i}+v_{t+1\;}^{i}$$

Based on the above, the following 10 features are available for each data record:

(9) $$\left(V_{x},V_{y},\;A_{x},A_{y},S_{x},S_{y},C_{x},C_{y},n_{\left(R_{a},R_{C}\;\right)},n_{\left(R_{s}\right)}\right),$$

The next section describes how we label these data records.

### 2.2 Labelling Collective Motion Features

The boid model described in the previous paragraph was simulated on a computer. Different combinations of weights, radius parameter values, and forces assigned to boids in a point mass simulator result in a new type of behaviour of boids with different formation patterns. The dataset includes 16 different parameter setup combinations to generate 16 behaviours. These parameter setups results in eight “structured behaviours (behaviours with an embedded identifiable pattern in the boids’ motion, as determined by the authors)” and eight “unstructured behaviours (random behaviours with no embedded pattern recognisable in the motion of boids)” using the investigations provided by Khan et al. [6]. Each behaviour is simulated in 200 point masses for 1501 timesteps of simulation. The motion generated forms a frame of the video for each of these timesteps. We record the full temporal state parameters of every boid in each video frame from the underlying simulation data. This temporal state forms the feature set of our data. This results in 1501 records for each of the 200 boids with 10 features for each behaviour.

The participants of the survey watch a video captured from these 1501 timesteps of each of the 16 behaviours. Each video is about 10 seconds, in which boids execute the same pattern of motion as in the 1501 simulated timesteps. To associate one of each of the three binary labels with a video (and thus with the corresponding 1501 × 200 records representing that video) the average response $\bar{V}$ of participants' survey answers is used, thresholded at 50% to generate a binary label. As participants gave three labels $\left\{L_{1},L_{2},L_{3}\right\}$ each of these was averaged permitting us to create three datasets (the Flocking Dataset, the Alignment Dataset, and the Grouping Dataset). Each data has a data structure the same as Fig. 1 for each of the 16 behaviours.

Each of the three datasets include 4,803,200 samples which is the combination of 1501 timesteps for 200 boids for 16 behaviours (1501×200=300200 for each behaviour and 300200×16=4,803,200 for the combination of all behaviours). The next section provides more information on how participant data was collected and averaged to compute labels.

## 3 Experimental Design, Materials and Methods

**Fig. 2:**
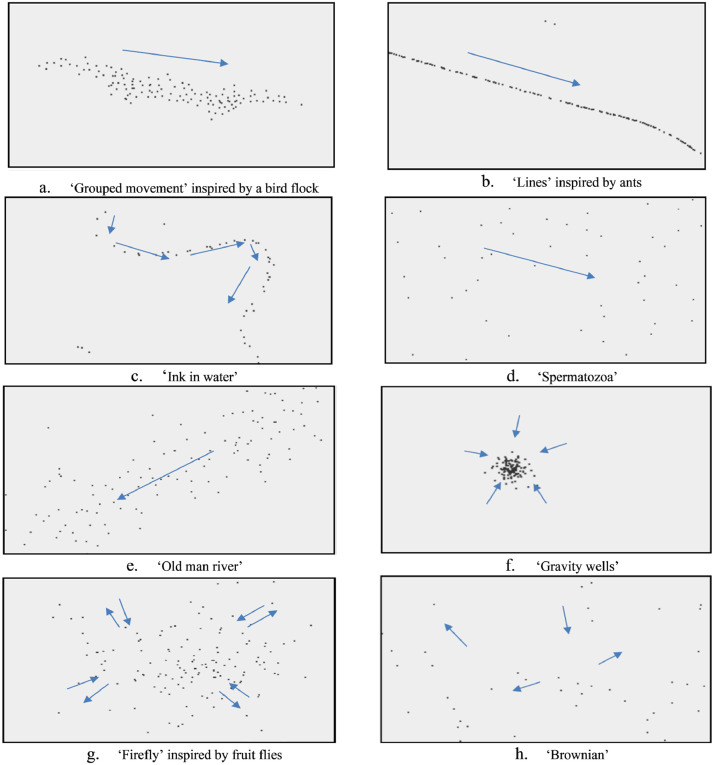
Eight, simulated, structured behaviours from [6].

Data on human perception of collective motion was collected using an online survey. The online survey includes 16 questions, which each represents a short video clip of the corresponding boids’ motion followed by three range sliders. The 16 behaviours include eight structured and eight unstructured behaviours. These 16 behaviours were introduced by Khan et al. [6] to provide a representative range of collective or random motions. The structured collective motions include boid movements with an identifiable embedded pattern, while unstructured collective motions consist of random movement of boids with no patterns. The eight structured collective motions are presented in Fig. 2.

In the online survey, three labelling scales were available to participants: flocking-not flocking; aligned-not aligned and grouped-not grouped. The term ‘flocking’ was chosen as a recognizable word from common vernacular that could be easily described in the relatively uncontrolled conditions of an online survey. The terms ‘aligned’ and ‘grouped’ were chosen as alternatives in case of limited agreement on the term ‘flocking’, following the methodology of Harvey et al. [12].

The online survey contained a tutorial phase and a question phase, which are shown in the supplementary materials. The tutorial phase was included to provide participants with a basic introduction to collective motion, specifically ‘flocking’ motion and the two related concepts of ‘alignment’ and ‘grouping’. This is achieved by showing two videos of natural collective motions and two videos of the simulated collective motions, accompanied by a small amount of descriptive text. Definitions of these three terms were also available as a ‘quick guide’ in the question phase.

The tutorial phase was followed by 16 ‘questions’ which form the question phase of the survey. Each question contained a short video (around 10 seconds = 1501 simulated timesteps), captured from the simulation of each of the 16 behaviours in point masses. The data records related to each of the 16 behaviours are for 1501 timesteps of 200 boids moving in a rectangular area of 1000×1400 pixels. Each of the 16 behaviours keeps the same embedded pattern of the motion within the 1501 timesteps. The short 10 seconds video clips shown to the participants are a visual representation of these 1501 timesteps, which helps the participant to identify the existence (or not) of the embedded pattern. The videos are shown in a randomized order to the participants, while the participants are not aware of these randomized orders. 90 participants answered these 16 questions. They were asked to give three opinions about each movement, regarding their perception of the grouping, alignment, and flocking nature of the movement. To this end, for each video, three range sliders were provided in the survey. The sliders allowed participants to record their responses on three scales for (1) flocking to not flocking; (2) aligned to not aligned; and (3) grouped to not grouped. The scales are from 0 to 100. The participants must put the slider point closer to the term which is most similar to their opinion of the presented video. Screenshots of the questions of the survey are provided in the supplementary materials, which also show the three range sliders.

In each question, the final slider position V is recorded as a value between 0 and 100. We consider each participant's answer as three parts $\left\{L_{1},L_{2},L_{3}\right\}$, where $L_{1}$ is the label from the first range slider (Flocking-Not Flocking), $L_{2}$ is the label from the second range slider (Aligned-Not Aligned), and $L_{3}\;$is the label from the third range slider (Grouped-Not Grouped). $L_{1}=1,\;L_{2}=1$ and $L_{3}=1$ if the extracted human perception is flocking, aligned, and grouped, respectively. The values assigned to the labels are equal to zero if the extracted human perception is not-flocking, not-aligned, and not-grouped. Since the slider position V is recorded as a value between 0 and 100, if the participant's answer is V<50, the behaviour is recognised as flocking, aligned, or grouped by the participant. Inversely, V>50 means that the behaviour is recognised as not-flocking, not-aligned, or not-grouped by the participants.

**Fig. 3:**
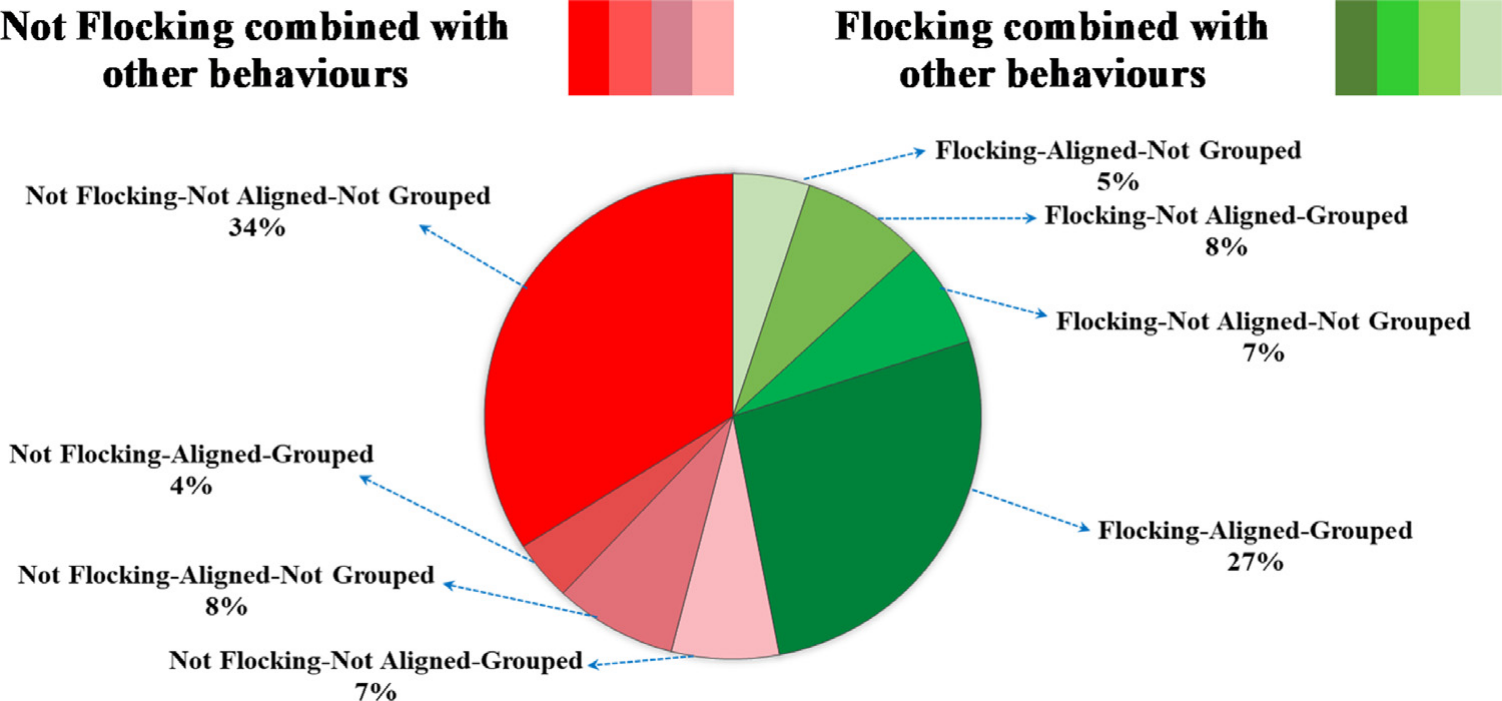
Percentage of participants’ responses in each combination of behavioural descriptors.

Before using this as the labelling scheme we inspected the combinations of participants’ responses by counting the number of answers fulfilling each combination condition, and then dividing this number by the total number of answers. Participant response combinations are visualized in Fig. 3. This figure shows that the most common label combinations are ‘Not flocking, Not aligned, Not grouped’ and ‘Flocking, Aligned, Grouped’ (the dark red and dark green sectors respectively). However, participants still report perceiving ‘flocking’ in the absence of one or the other, or even both alignment and grouping. Likewise, they will report perceiving ‘not flocking’ in the presence of grouping and alignment in a small number of cases. This indicates that there are individual differences in perception of flocking. In addition, this approach identified approximately 12% of responses had V=50. We discarded all data records with V=50from the dataset before considering an alternative labelling strategy.

**Fig. 4:**
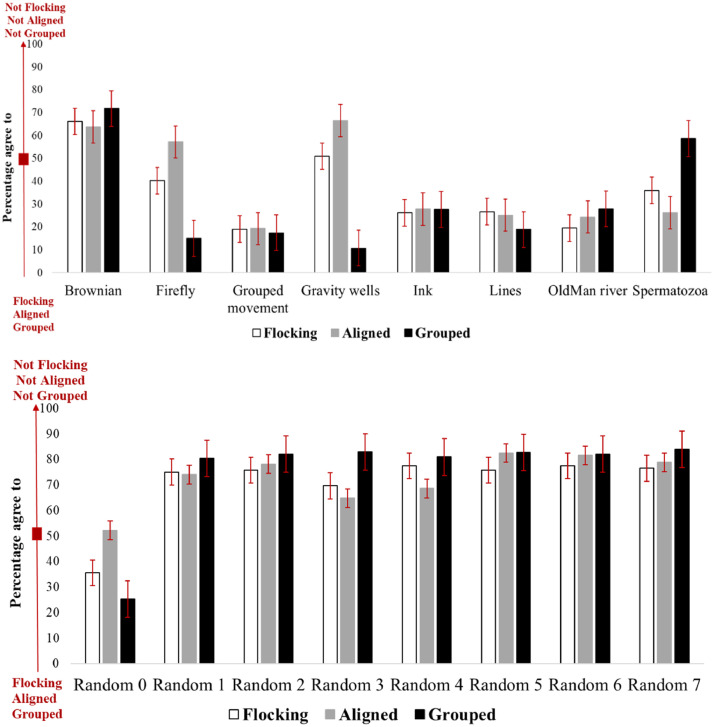
Human perception (n=90) of flocking, alignment and grouping for different boid behaviours. Error bars show the 95% confidence interval.

Given the individual differences in responses observed above, we then analysed average responses to examine whether this might be a more reliable way to label our data. In this approach, participant answers are again interpreted as values between 0 and 100 (V∈[0,100]). Then for each behaviour, the average of V values captured from the participants’ responses are computed, as $\bar{V}$, and $\bar{V}\in \left[0,100\right].\;$In this approach, V values are averaged across all participants Fig. 4 summarizes the average responses of 90 participants to the survey using this approach, including a 95% confidence interval. The red arrow in the left part of this figure represents the interpretation of the average values $\bar{V}$, which has the same meaning as the range sliders described above. We see that there is a statistically significant difference between participants’ responses to a majority of the structured behaviours, compared to their responses to the unstructured behaviours. Participants reliably labelled several different types of motions as flocking ($\bar{V}<50$). These labels are then used to label each corresponding data record as described in the data structure section. We thus select this approach to create labels for our dataset. Hence, based on the corresponding $\bar{V}$ of the red arrow, again the behaviours with $\bar{V}<50$ are labelled as flocking, aligned, or grouped with the label value of 1. Also, the behaviours with $\bar{V}>50$ are labelled as not-flocking, not-aligned, or not-grouped with the label value of 0. Based on Fig. 4, in this approach by using the average perception of participants, $\bar{V}=50$ never occurred, and therefore no samples were required to be discarded from the data. The final step was then to confirm that these labels can be learned accurately by a machine.

**Fig. 5:**
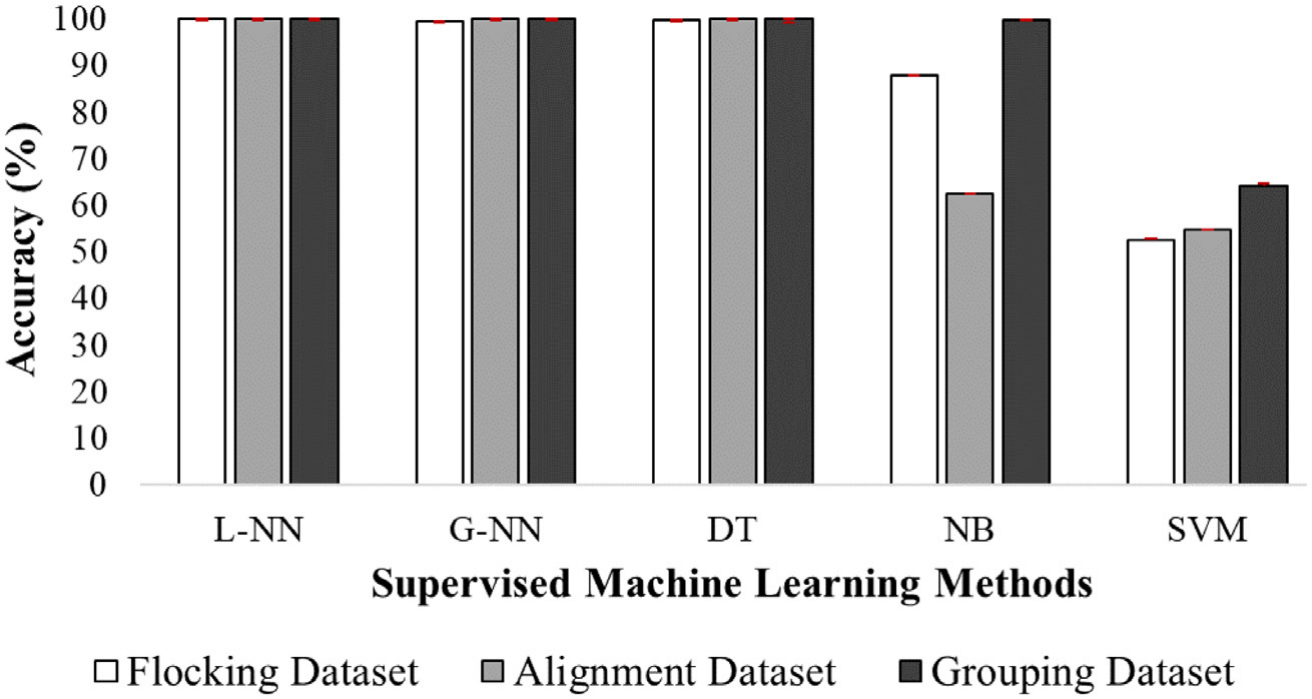
Average accuracy over 30 runs of 10-fold cross validation of supervised learning methods with 95% confidence interval.

To confirm our data is suitable for a supervised machine learning algorithm to learn from, we trained five different machine learning methods with our data. The methods used are Levenberg Neural Network (L-NN) [13], Gradient Neural Network (G-NN) [13], Decision Tree (DT) [14], Naïve Bayes (NB) [14] and Support Vector Machine (SVM) [14]. The parameter setup for these machin.e learning methods is based on the default parameters of MATLAB, and the number of epochs for the neural network is 10. The five supervised machine learning methods are trained with each of the binary datasets (Flocking, Alignment, and Grouping). The training performance of machine learning models on the proposed data could be evaluated by different protocols including k-fold cross-validation, leave-one-out and splitting the data into train and test samples. However, since evaluating the performance of machine learning models is not the main goal of this paper, the following experiment only considers the 10-fold cross-validation. Cross-validation is a common method to evaluate machine learning models without being affected by over-fitting. Therefore, to prevent over-fitting of the supervised methods, 30 runs of 10-fold cross-validation [15] were applied. Fig. 5 shows the average accuracy of the five supervised learning methods, over each of the 10 folds, with a 95% confidence interval (the same results with 98% of confidence interval are discussed in Section 3 of the supplementary material). Also, Table 1 shows the processing time of these supervised methods. As presented in this figure, three of the supervised methods accurately learned to label the data according to the human perception labels, including flocking, alignment and grouping criteria. Among them, DT achieves the highest accuracy and lowest processing time. This result confirms that our chosen labelling strategy is appropriate for a machine learner to automatically recognise collective behaviour, providing an appropriate machine learning algorithm is used. These machine learning models are further investigated by applying leave-one-out and train/test data split protocol in Section 2 of the supplementary material.

**Table 1:** Processing Time in seconds of supervised learning methods with 95% confidence interval.

	Flocking Data	Alignment Data	Grouping Data
DT	5.15±0.03	5.31±0.04	5.49±0.03
NB	5.24±0.08	5.79±0.02	5.54±0.02
SVM	499.00±0.62	452.22±0.47	475.12±0.83
L-NN	1050.20±0.89	1050.20±0.89	1050±0.89
G-NN	90.00±0.25	92.19±1.45	90±0.25

## Ethics Statements

This study was carried out in accordance with the approval of the UNSW Canberra Human Research Ethics Advisory Panel with written informed consent from all subjects. The protocol was approved by the UNSW Canberra Human Research Ethics Advisory Panel HREAP HC190986.

## CRediT authorship contribution statement

**Shadi Abpeikar:** Conceptualization, Methodology, Writing – original draft, Visualization, Investigation. **Kathryn Kasmarik:** Supervision, Writing – review & editing.

## Declaration of Competing Interest

The authors declare that they have no known competing financial interests or personal relationships that could have appeared to influence the work reported in this paper.

## Data Availability

Replication Data for: Automatic Collective Behaviour Recognition (Original data) (Dataverse).

## Appendix Supplementary materials

Image, application 1
