# Supplementary material for: Motion behaviour recognition dataset collected from human perception of collective motion behaviour
Source: Data Brief. 2023 Feb 15;47:108976. doi: 10.1016/j.dib.2023.108976 (PMC9975684; doi:10.1016/j.dib.2023.108976)
Supplement: Supplementary file 1 [file mmc1.docx]

**Supplementary Material for: Motion Behaviour Recognition Dataset Collected from Human Perception of Collective Motion Behaviour**

**Section 1: Screenshots captured from the online survey.**


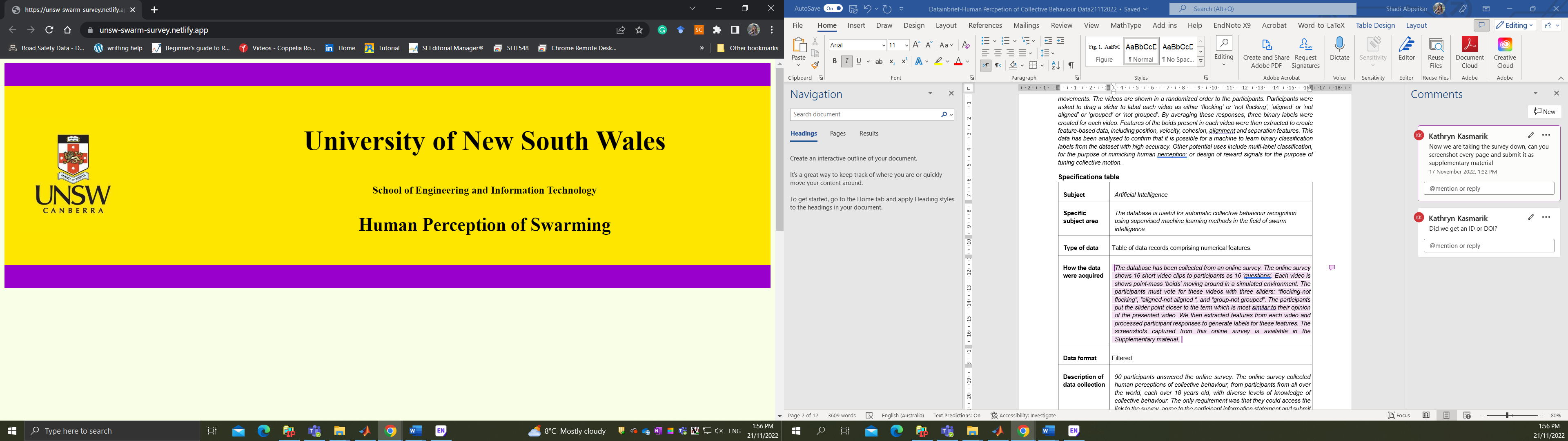

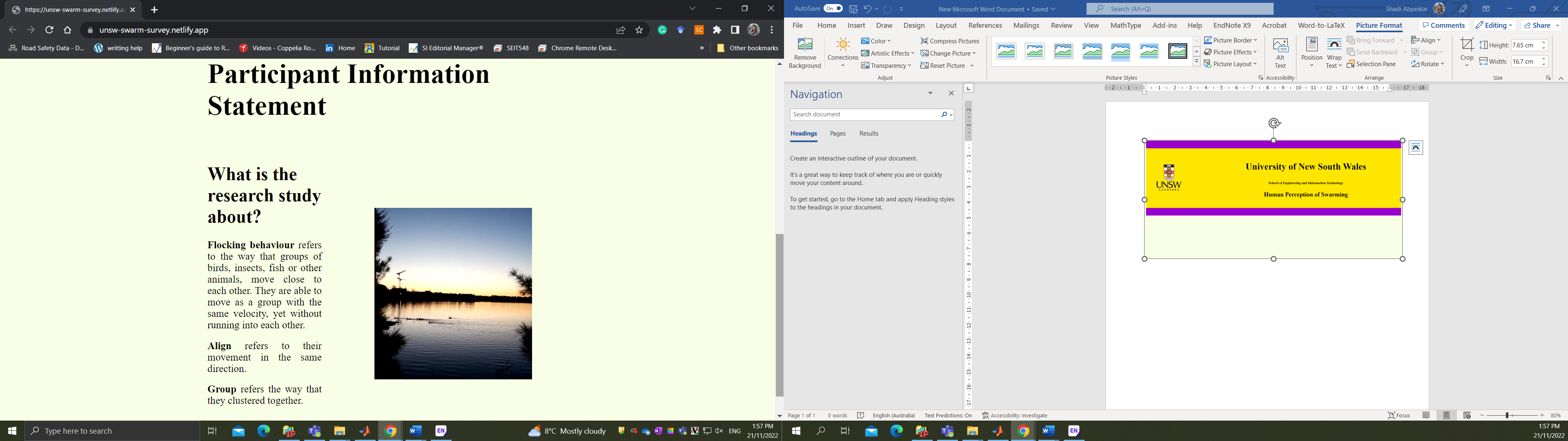

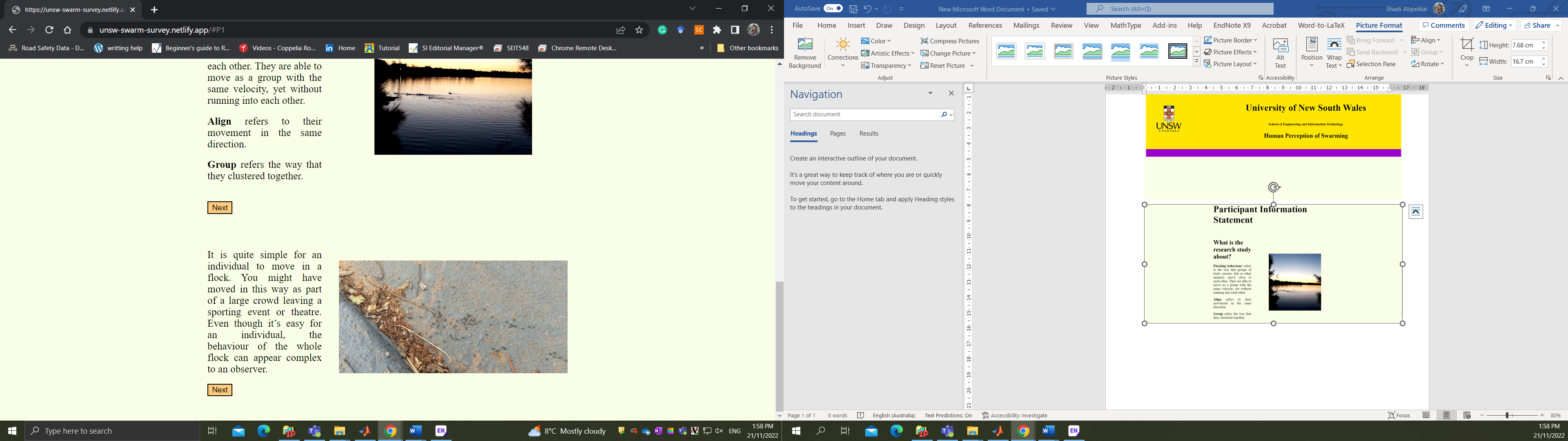

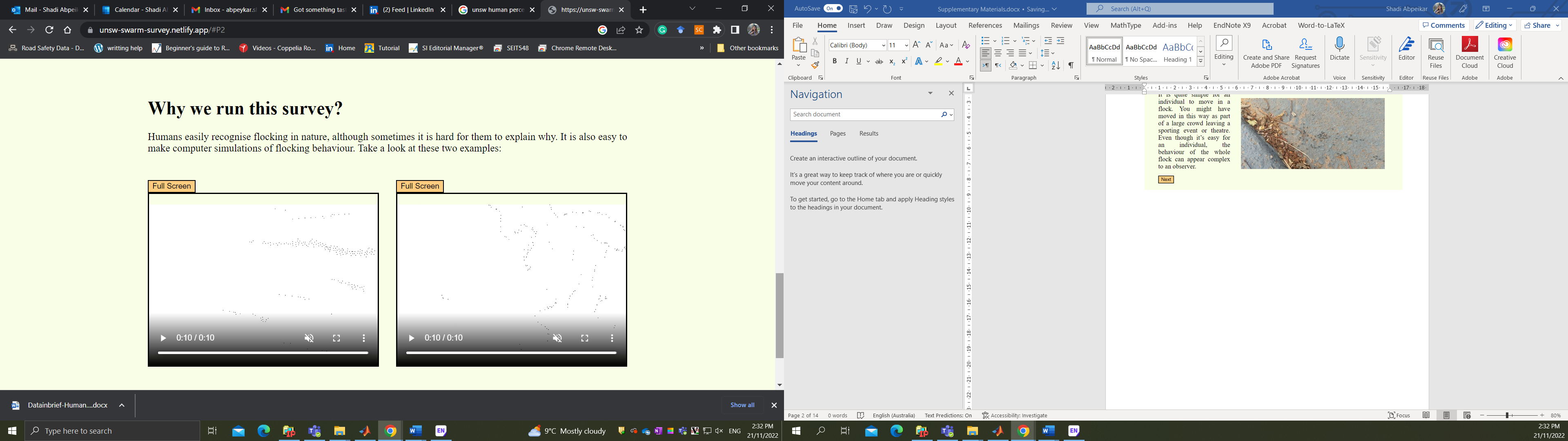

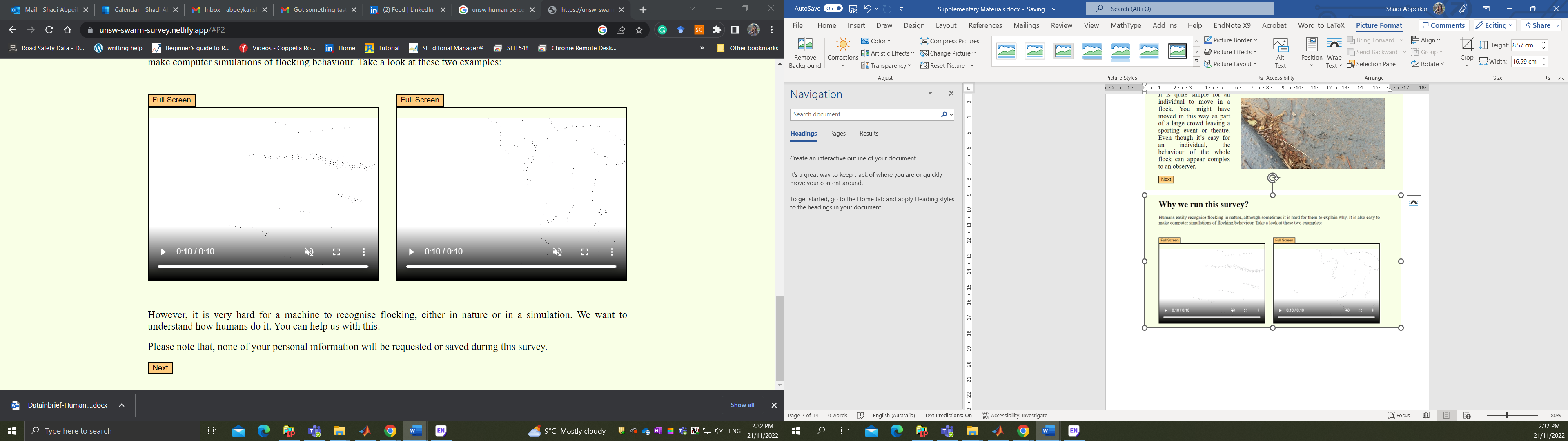


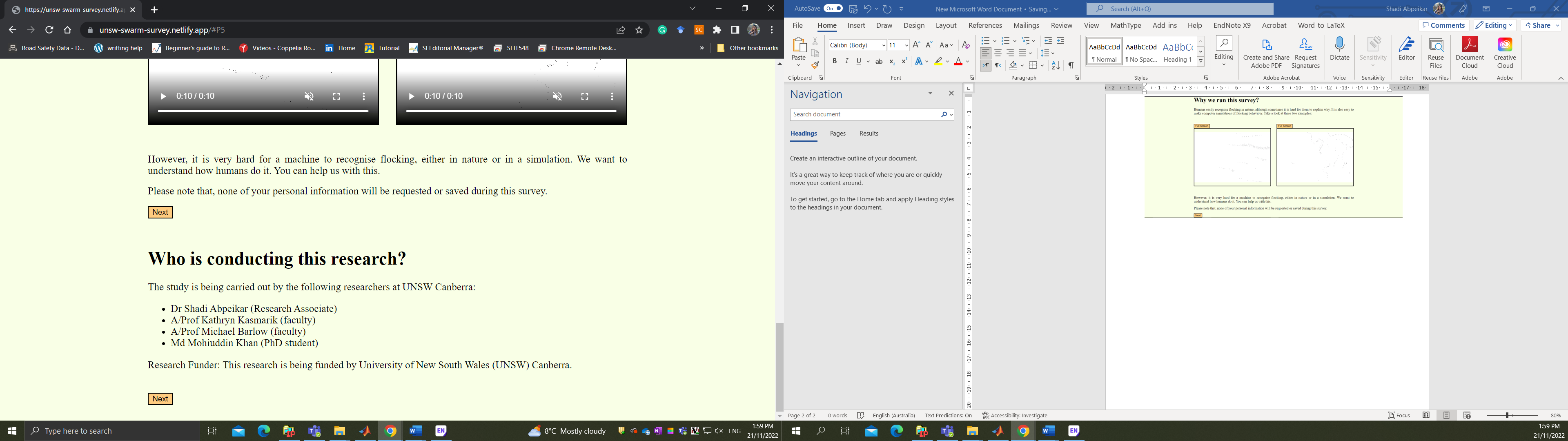

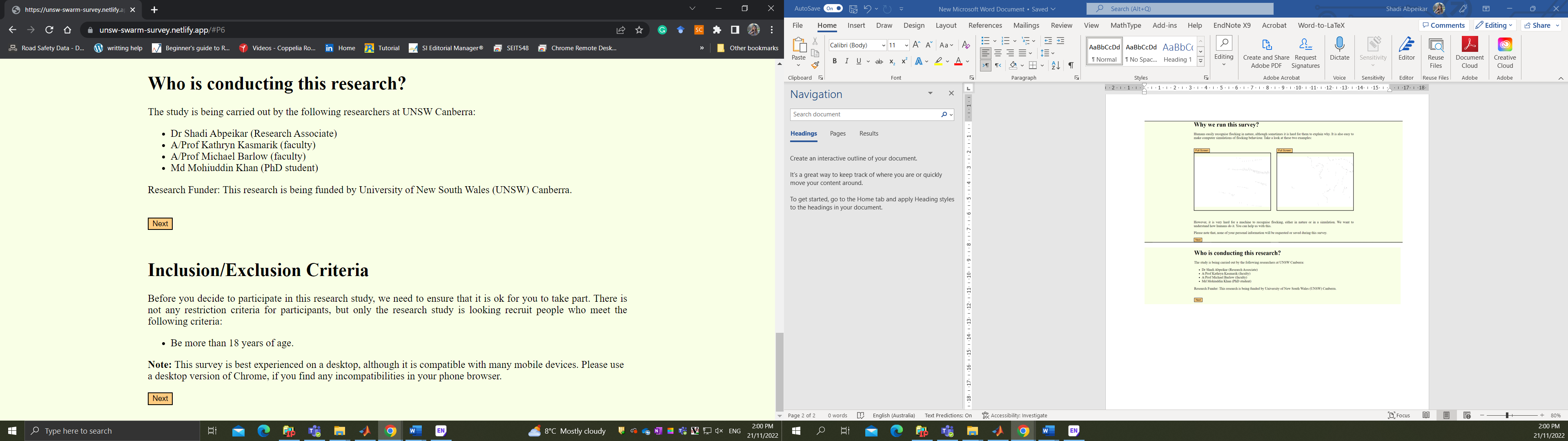

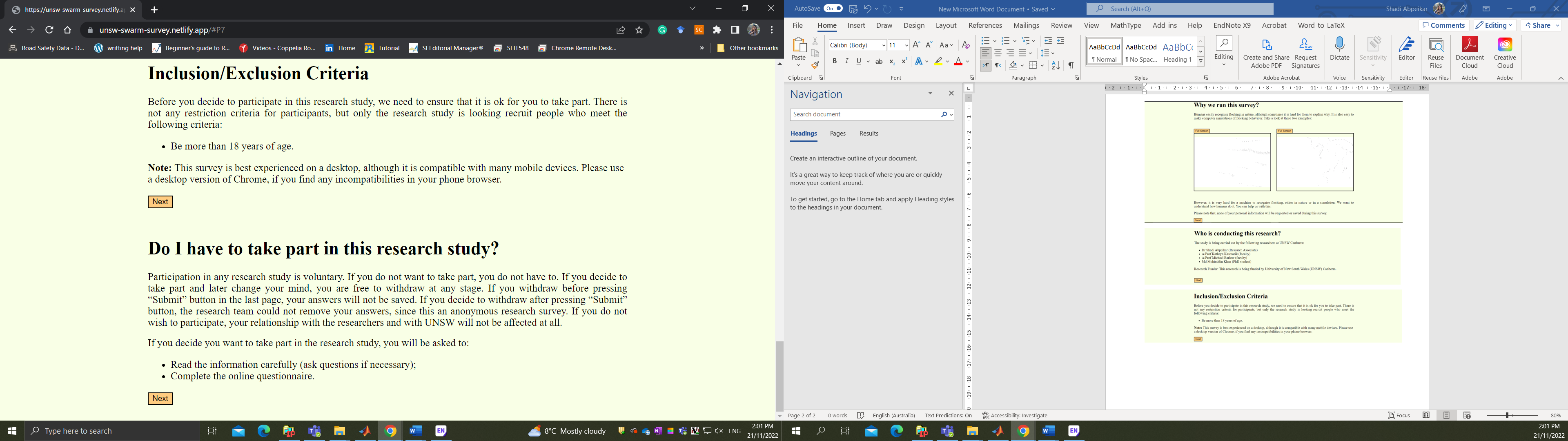


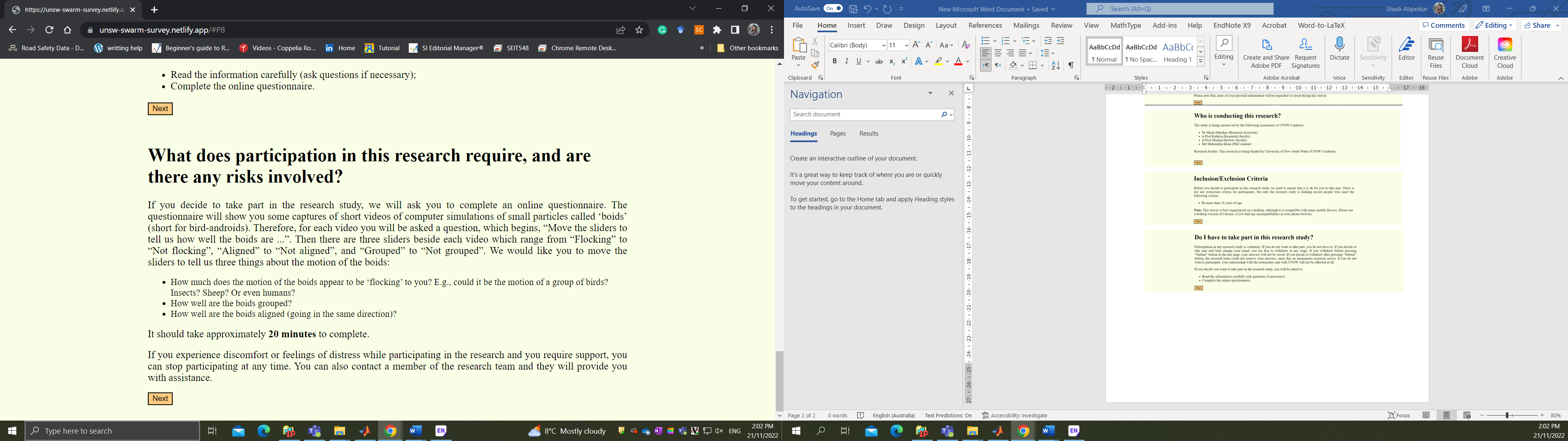

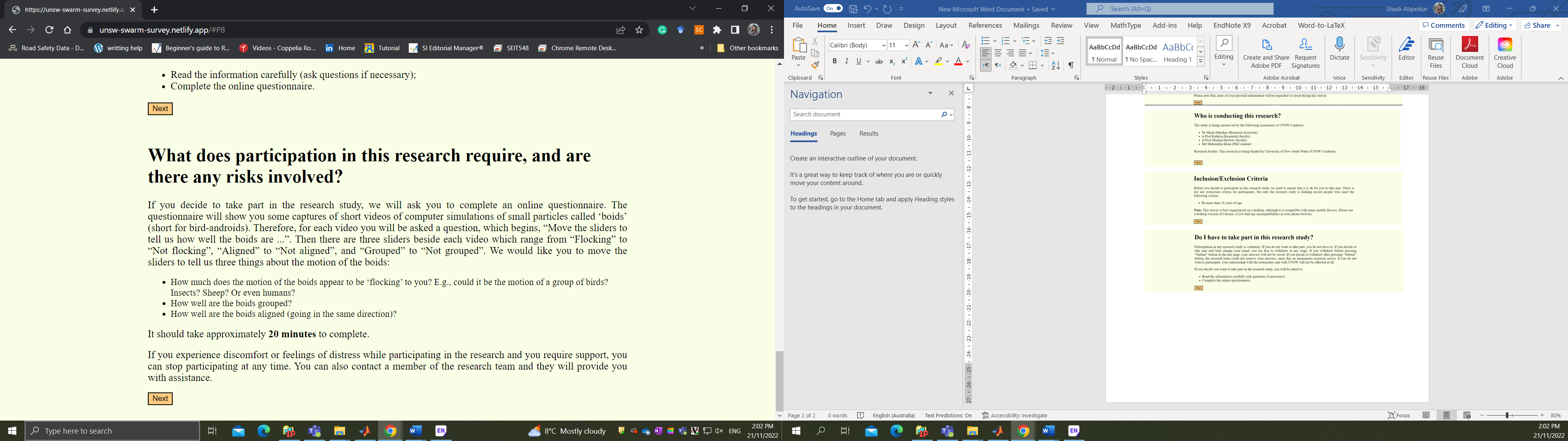


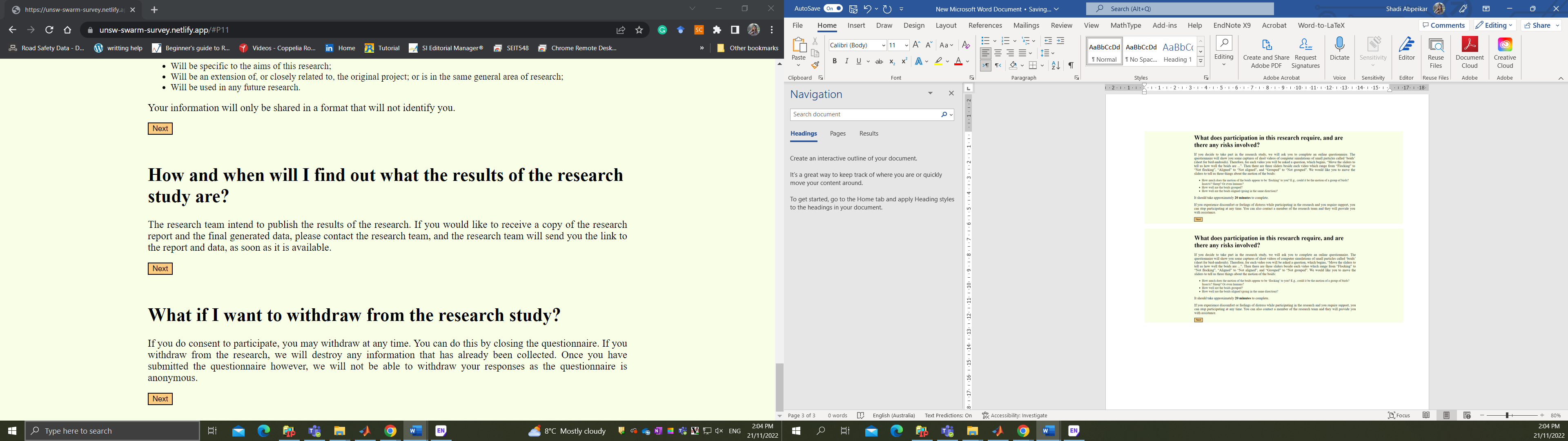

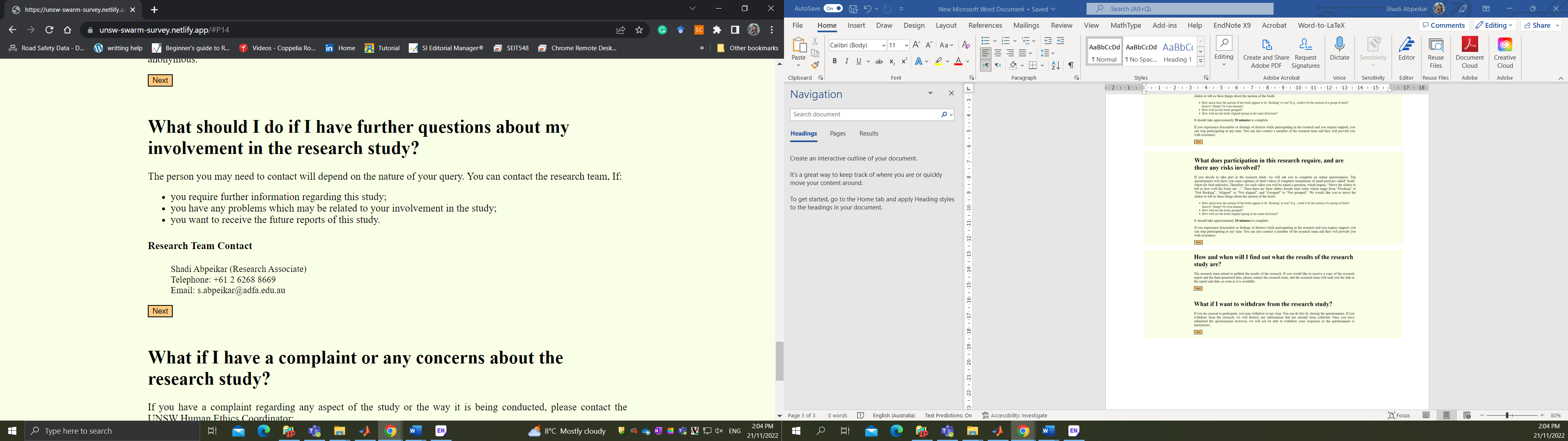


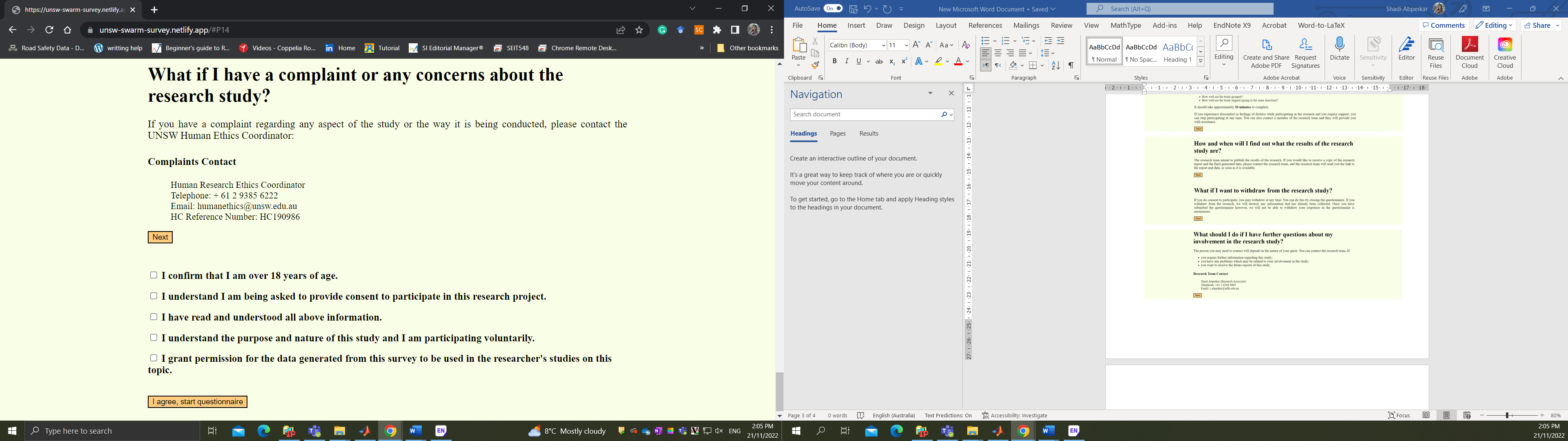

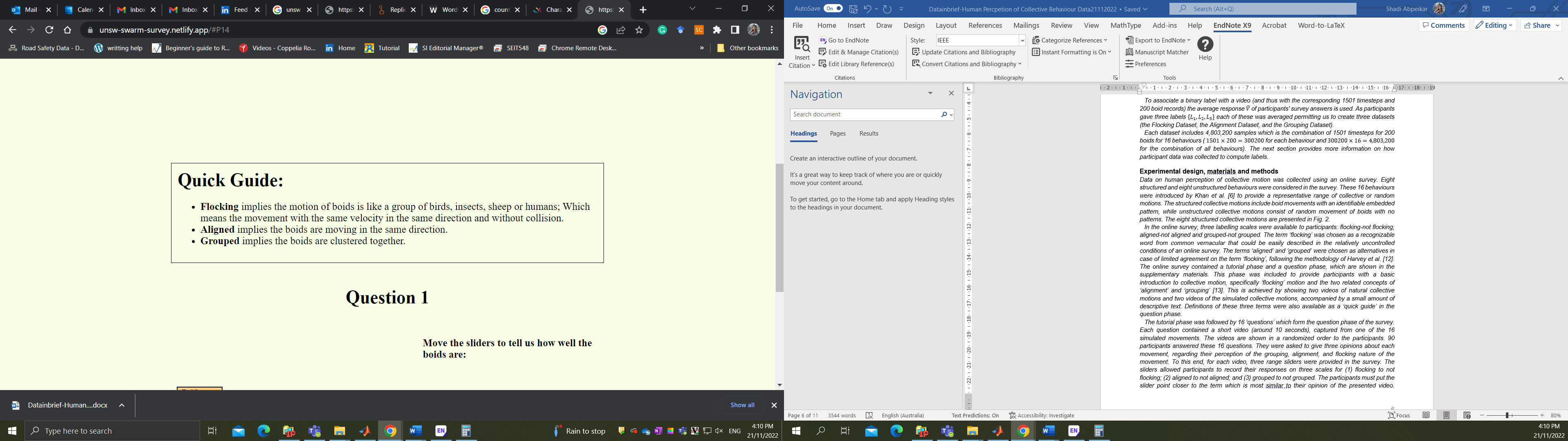

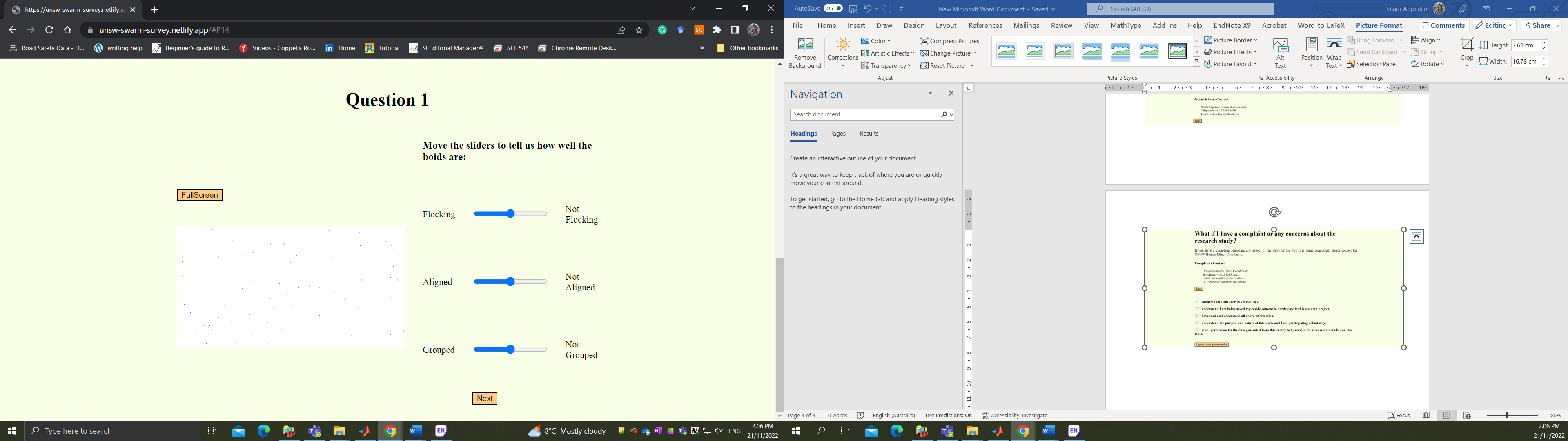


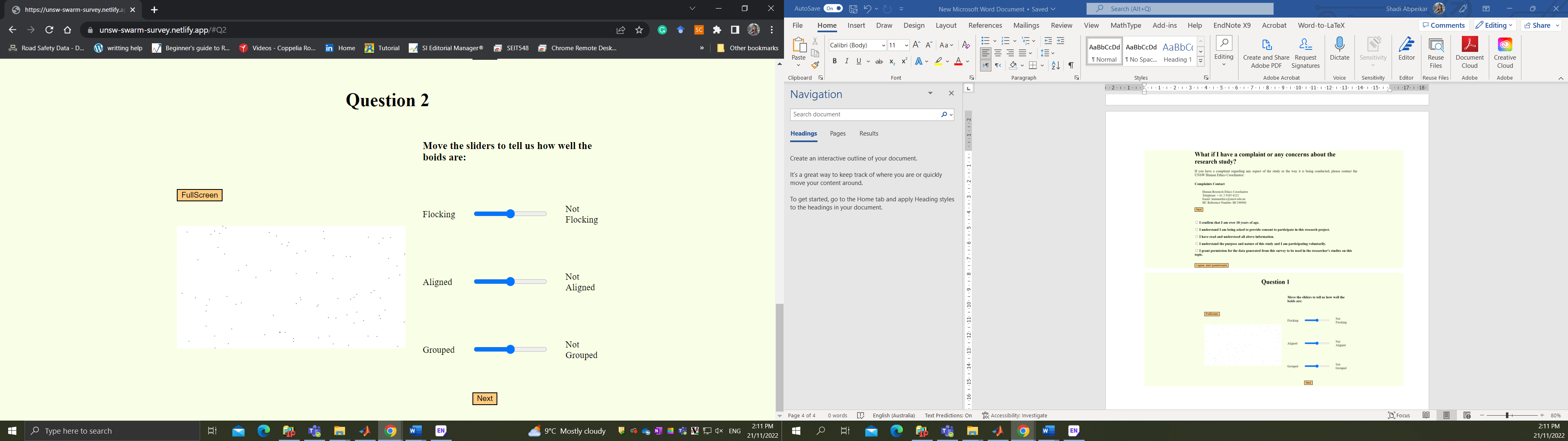

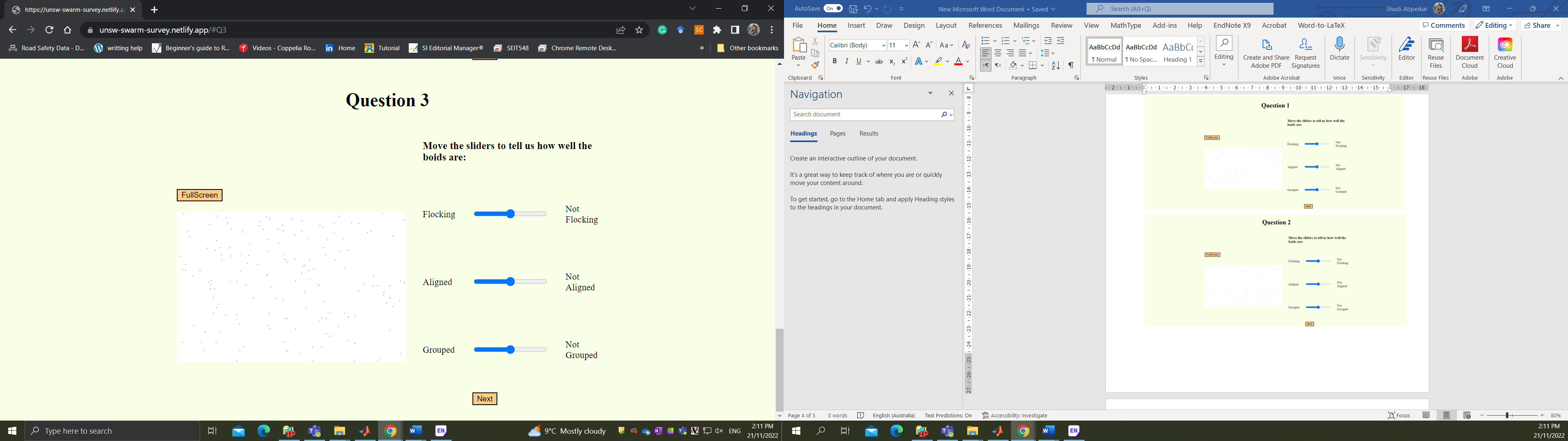


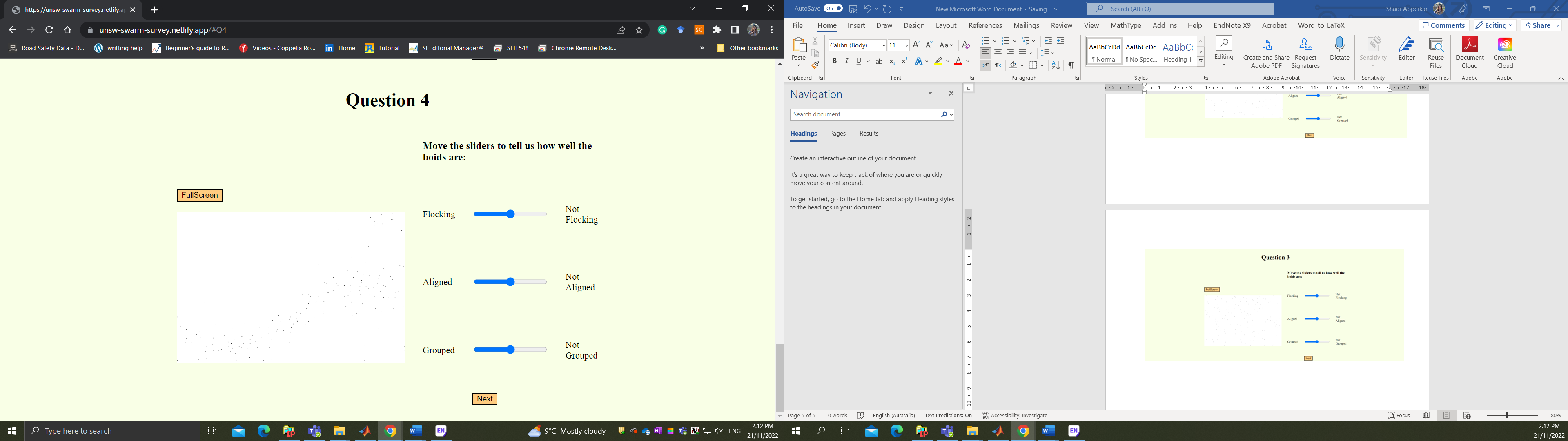

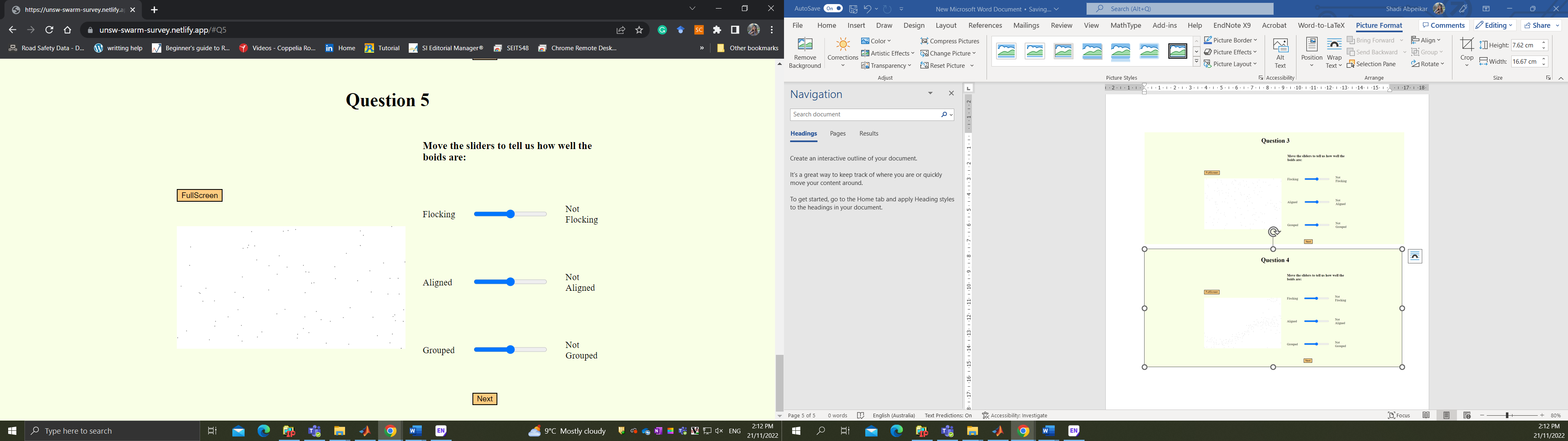


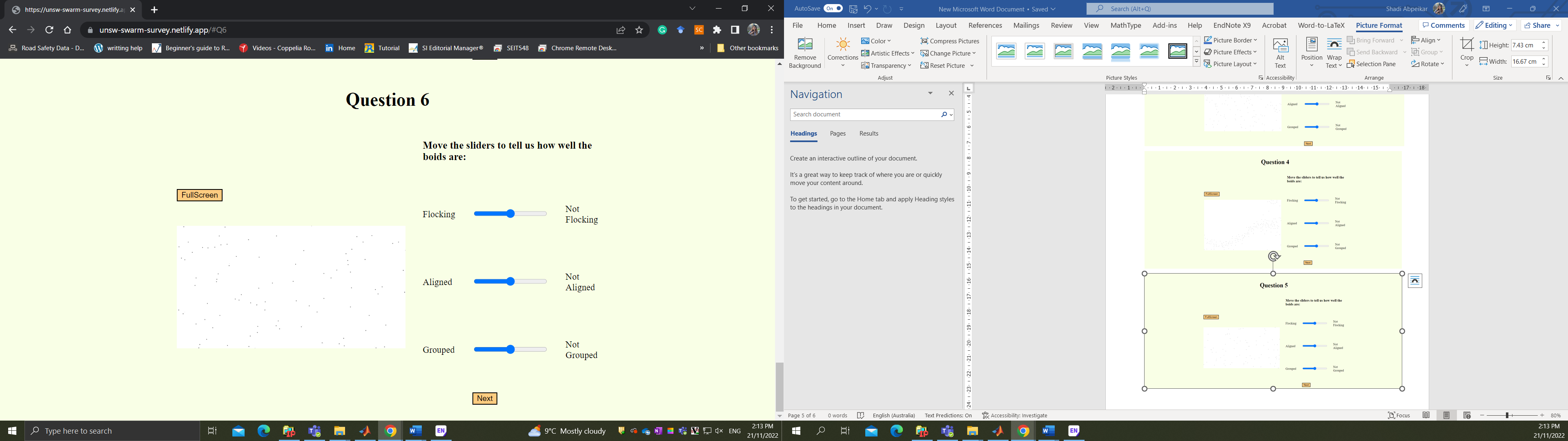

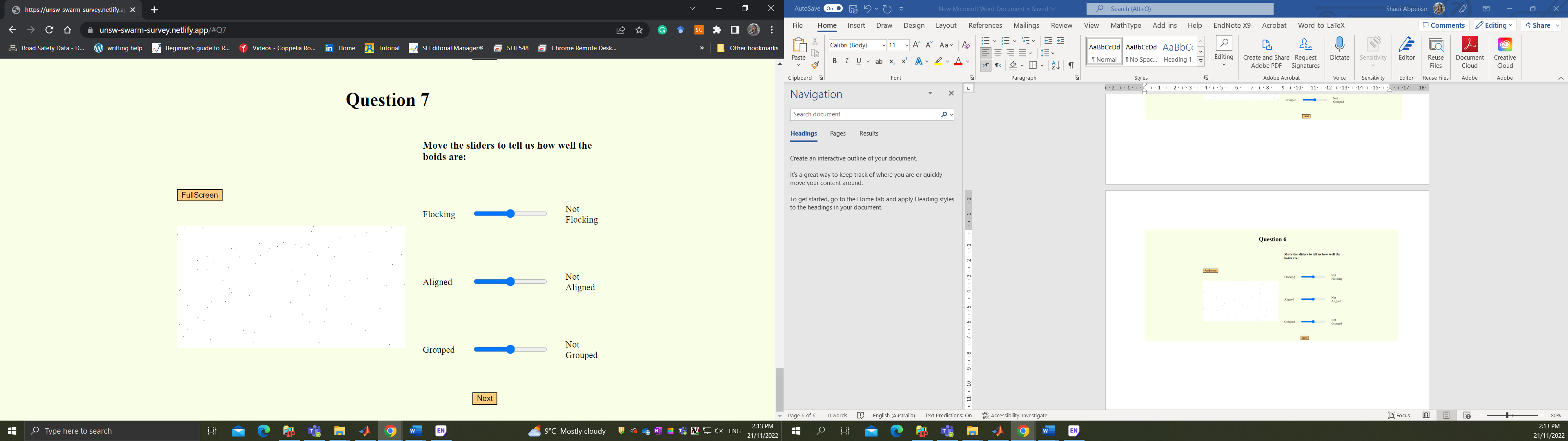


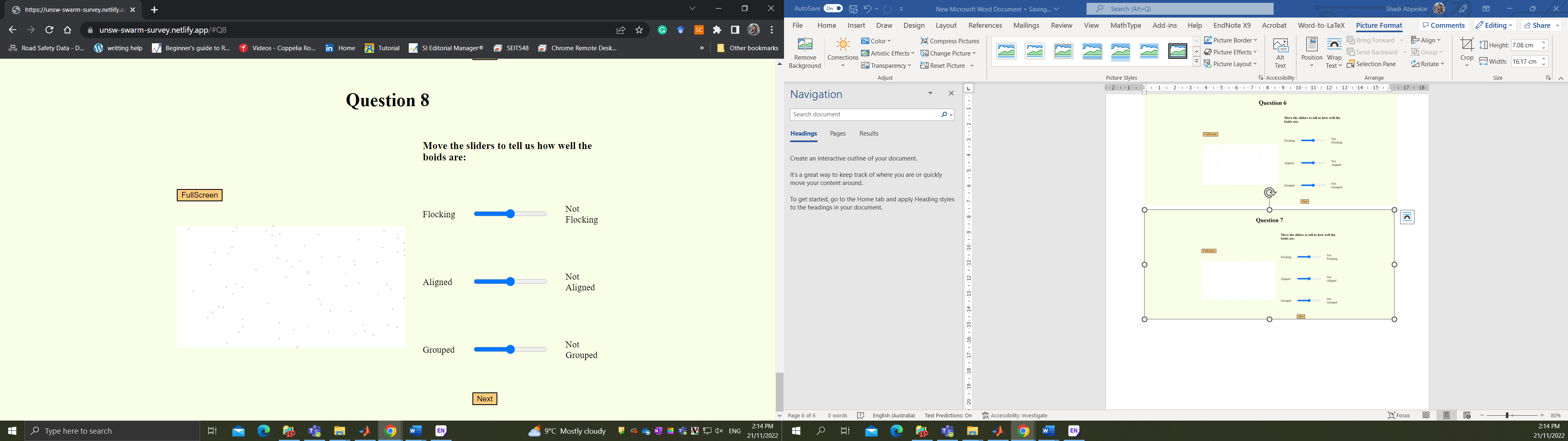

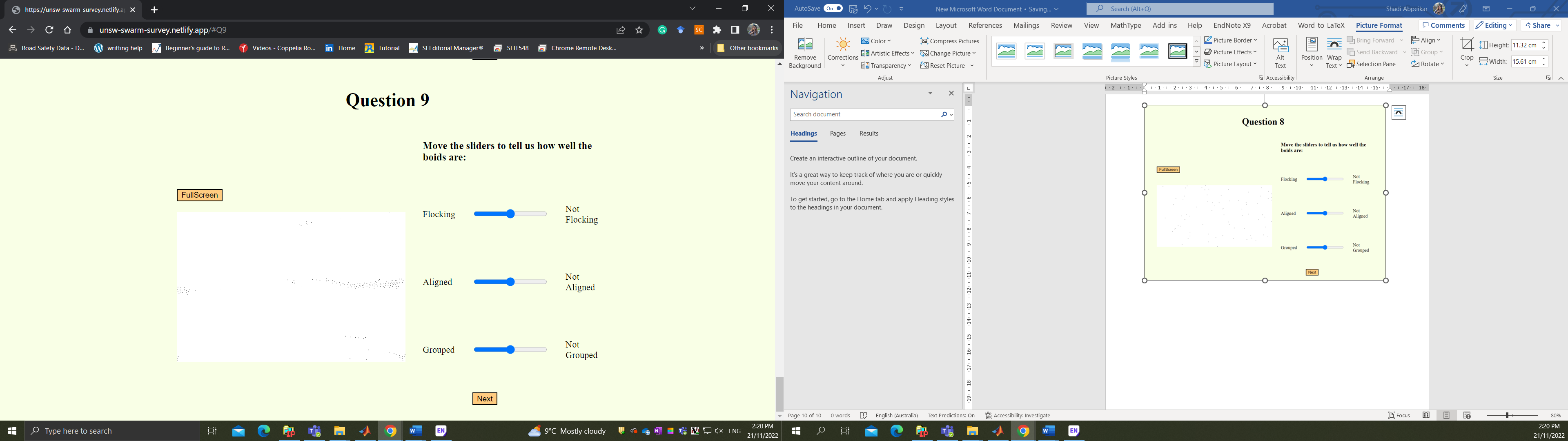


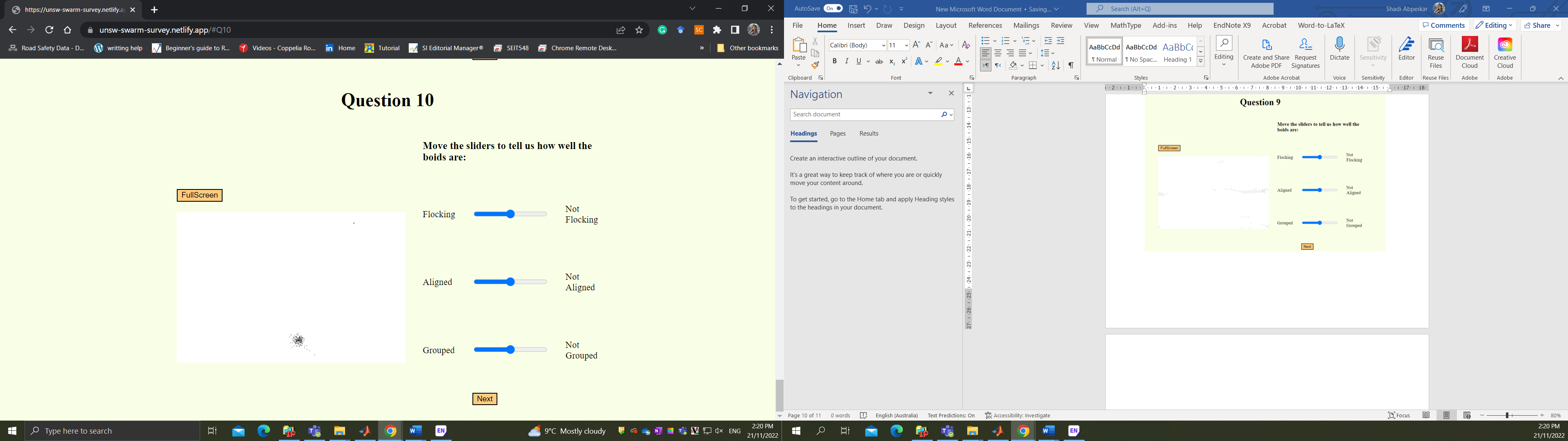

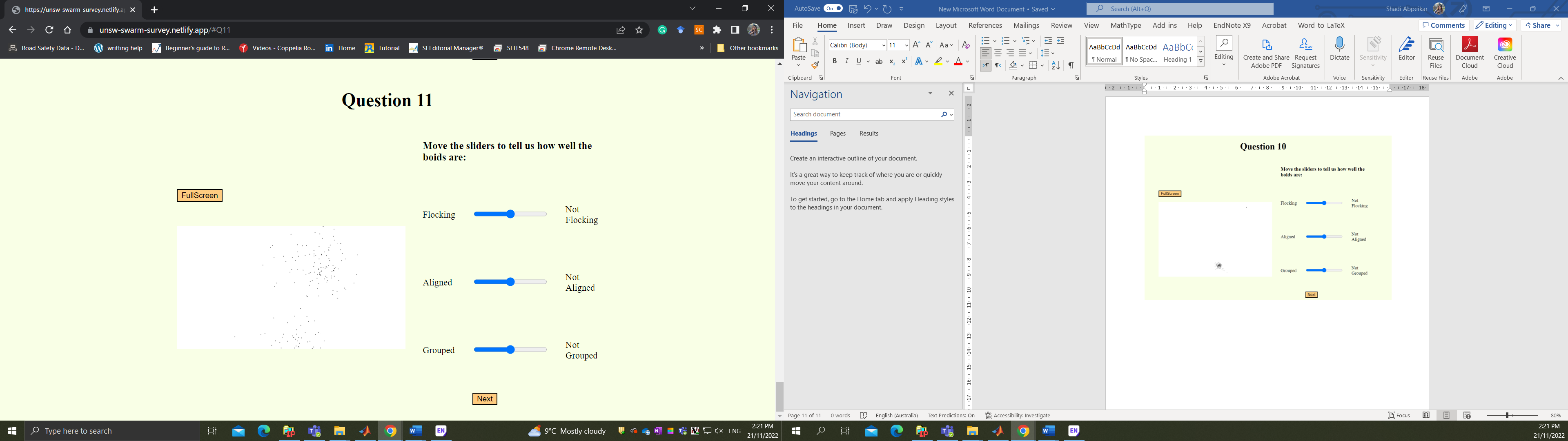


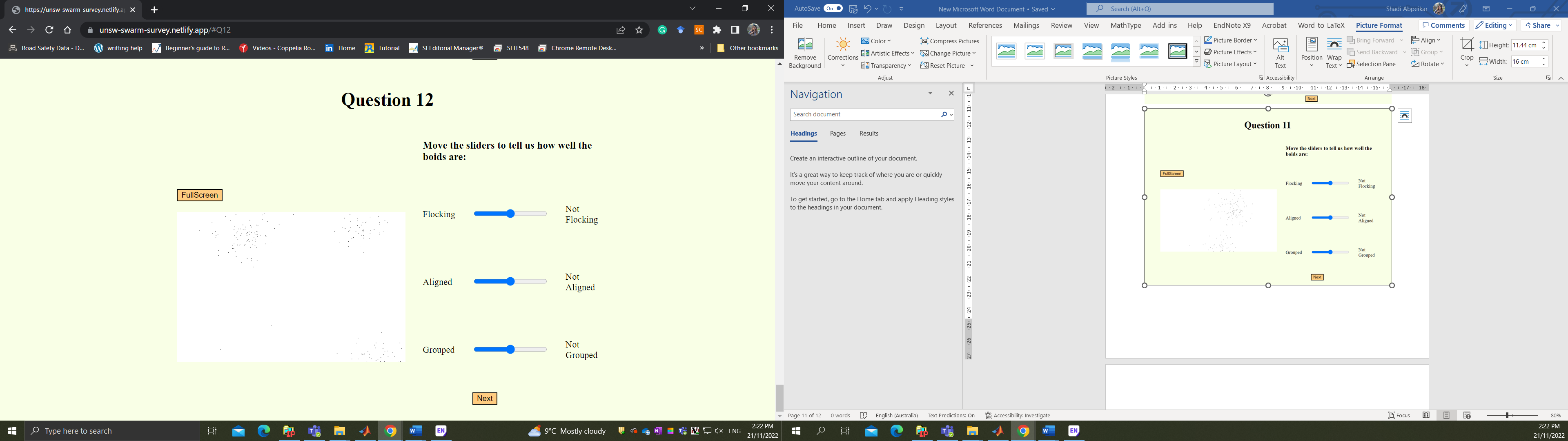

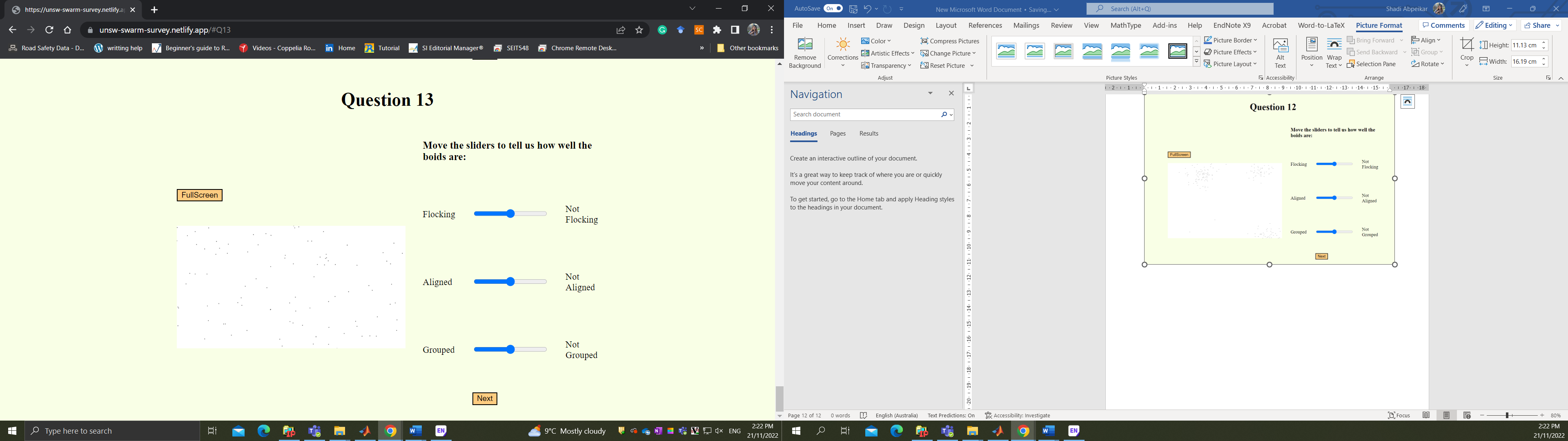


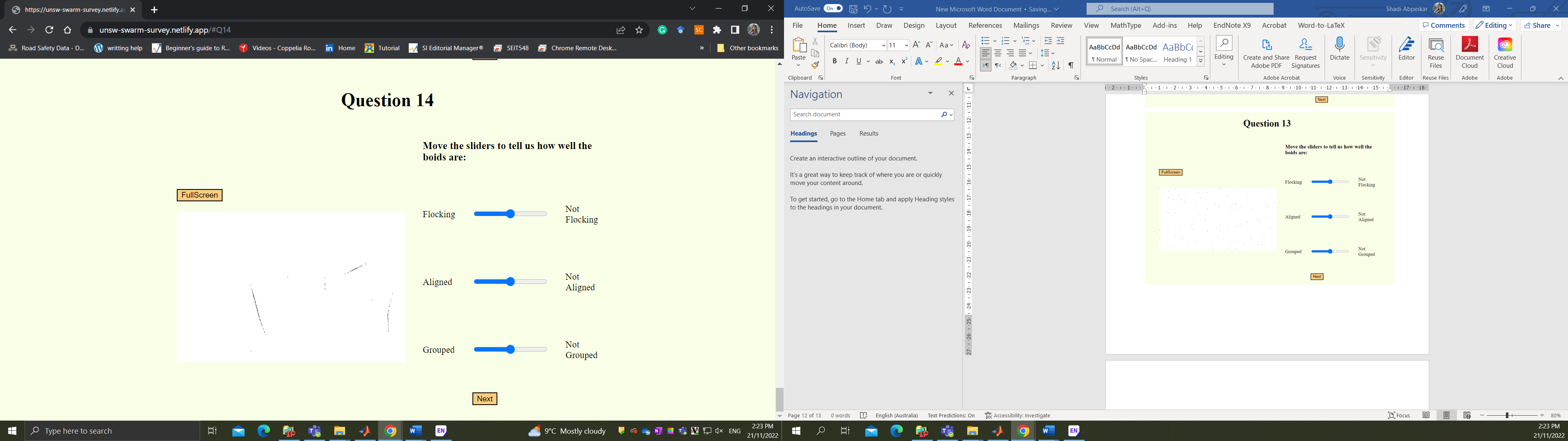

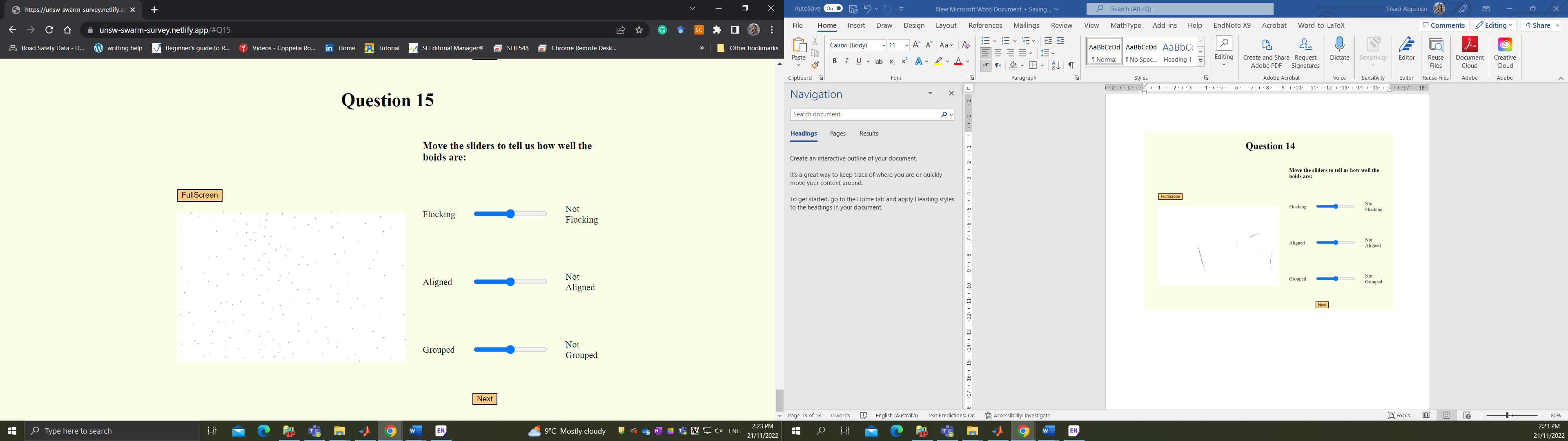


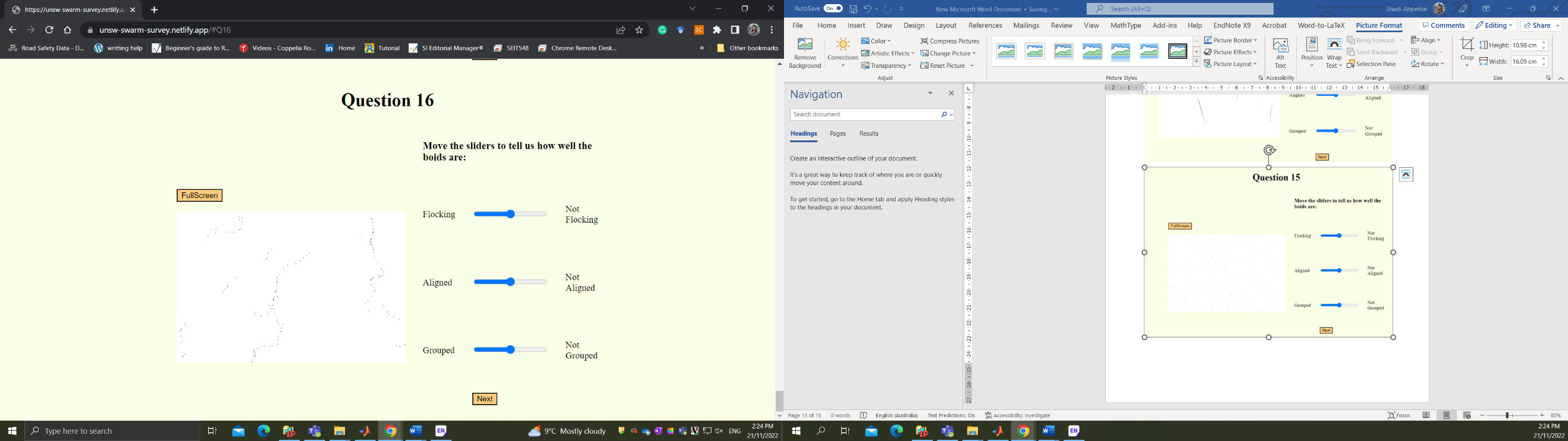

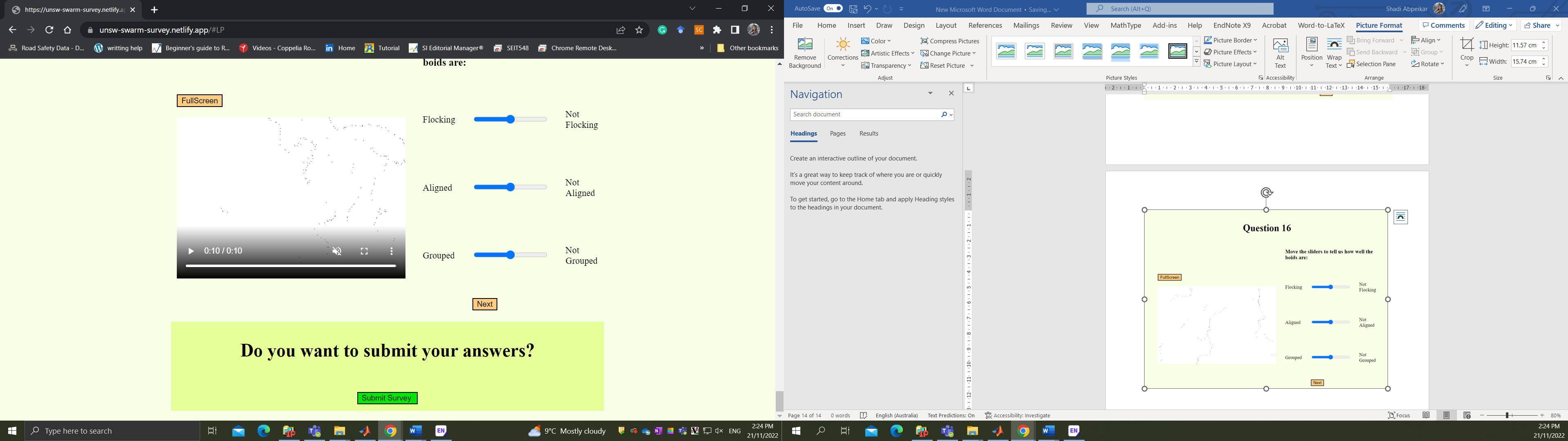


**Section 2: Performance of training machine learning modes based on leave-one-out and train/test data split protocol**

In this section, the performance of the five machine learning methods trained with the human perception data has been considered using two different protocols of leave-one-out and train/test data split. There might be other protocols to evaluate this experiment, which is out of the scope of this paper but could be investigated by interested readers. Also, there are some other metrics including F-Score, Recall, ROC, and Precision to evaluate the performance of machine learning models. This paper only focuses on accuracy. The two protocols are investigated as follows:

- Leave-One-Out protocol: In this experiment 30 runs of machine learning methods are applied by leaving out each of the behaviours of Fig. 2 of the main manuscript and the random behaviour from the training set and testing the data on the unseen left-out behaviour. In this experiment, the 30 runs are only applied to the flocking dataset. ‎Fig. S1 represents the average accuracy over these 30 runs with a 95% of the confidence interval. As presented in this figure, the training performance of the machine learning methods could be evaluated using the leave-one-out protocol. On the other hand, the accuracy achieved with this protocol is not exactly the same as the 10-fold cross-validation investigated in the main manuscript, as it was expected. However, still, the decision tree is the most accurate machine learning method.


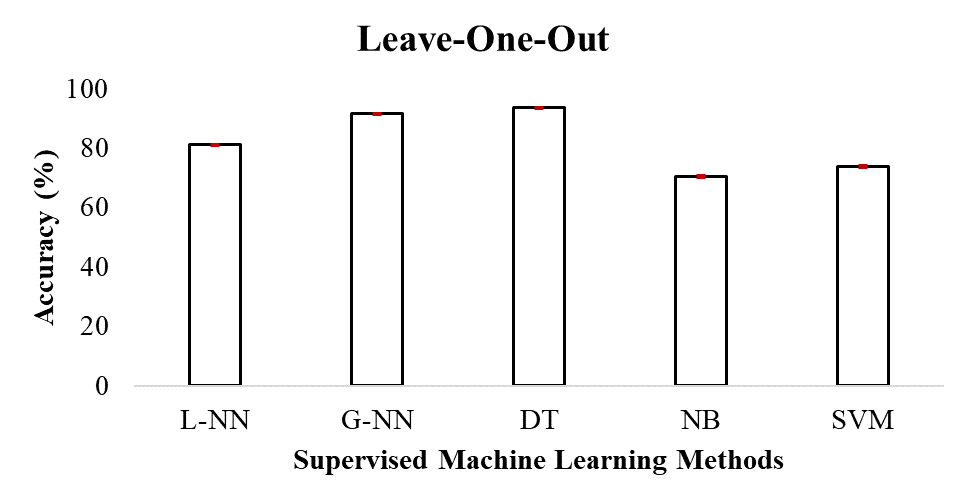


1. **Average accuracy over 30 runs of leave-one-out of supervised learning methods on flocking data with 95% confidence interval**

- Train/Test data split protocol: In this experiment 30 runs of machine learning models are investigated using randomly selected 30% of the data as the test data, and the remaining 70% as the training data. The average accuracy over test samples of these 30 runs with 95% of confidence intervals is presented in ‎‎Fig. S2. Again, as was expected the performance is not exactly the same as Fig. 5 of the main manuscript. However, still, the decision tree is the model which achieves high accuracy besides the L-NN, and G-NN.


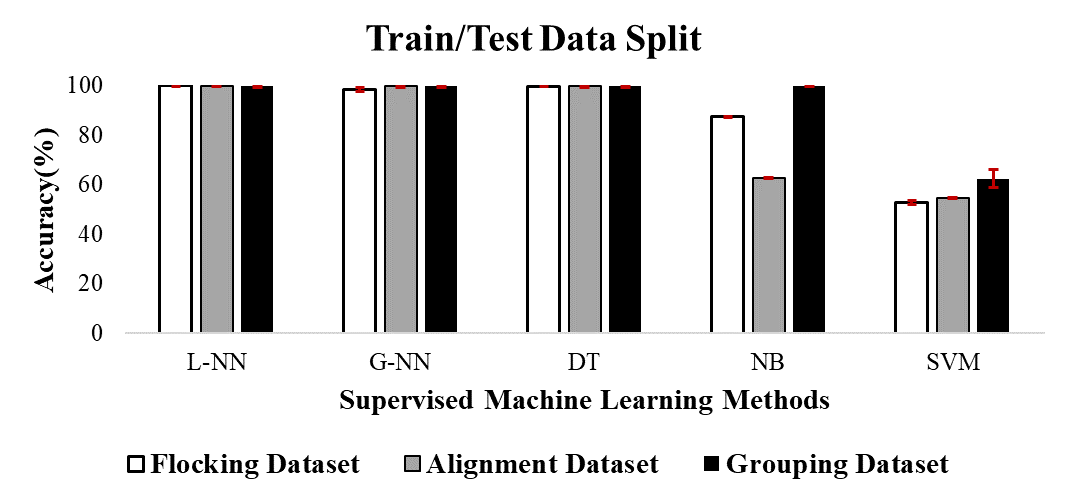


1. **Average accuracy over 30 runs of train/test split data of supervised learning methods with 95% confidence interval**

**Section 3: Performance of training machine learning models based on 10-fold cross-validation with 98% confidence intervals**

The results provided in Fig. 5 of the manuscript are with 95% of confidence intervals. The error bars provided with the green colour in ‎Fig. S3, are for the same experiment but with a 98% confidence interval. As presented in this figure, there is no large overlap between the confidence interval results.


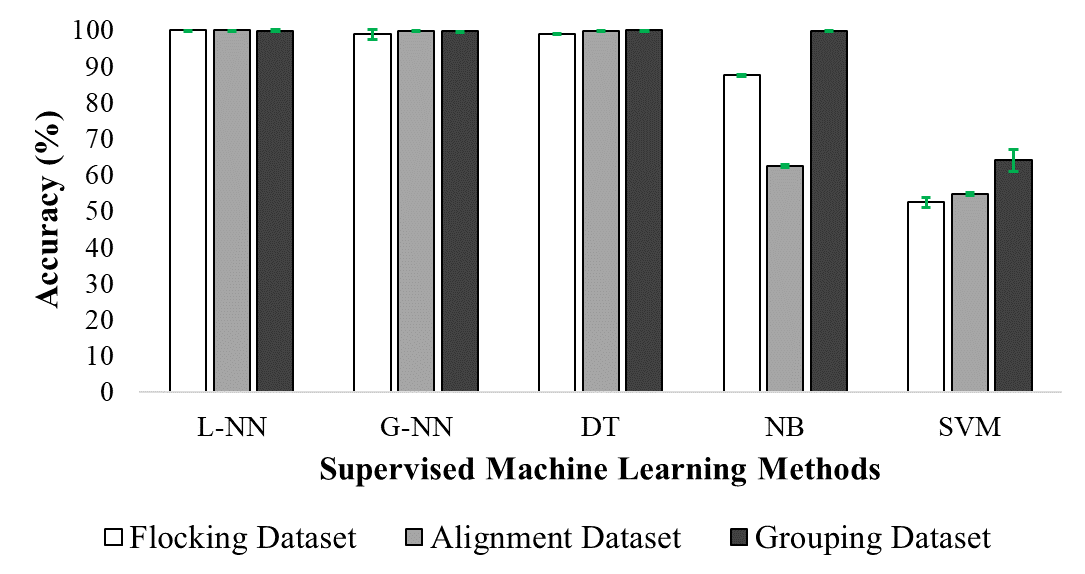


1. **Average accuracy over 30 runs of 10-fold cross validation of supervised learning methods with 98% confidence interval**
